# Soil Nutrient Dynamics and Fungal Community Shifts Drive the Degradation of *Pinus sylvestris* var. *mongholica* Plantations in the Loess Plateau

**DOI:** 10.3390/plants14091309

**Published:** 2025-04-26

**Authors:** Jiaxing Wang, Xiaotian Su, Yimou Luo, Yue Zhang, Yihan Wang, Jing Gao, Defu Wang

**Affiliations:** 1 College of Life Sciences, Shanxi Agricultural University, Jinzhong 030801, China; wjxfungi@sxau.edu.cn (J.W.); suxiaotian2023@163.com (X.S.); l15035907798@163.com (Y.L.); yuezhang0717@outlook.com (Y.Z.); 2 College of Tropical Crops, Yunnan Agricultural University, Pu’er 665099, China; 2023052@ynau.edu.cn

**Keywords:** *Pinus sylvestris*, soil nutrients, fungal community, plantation degradation, Loess Plateau, high-throughput sequencing, available potassium

## Abstract

The degradation of *Pinus sylvestris* var. *mongholica* plantations in Youyu County on the Loess Plateau has caused major ecological issues, though the mechanisms remain poorly understood. This study explores the effects of stand age and soil properties on the rhizosphere fungal community and their potential roles in plantation degradation. Soil samples were collected from plantations of different stand ages (13, 20, 25, and 35 years), and their fungal diversity and composition were analyzed using high-throughput sequencing. The results showed that soil organic carbon and total nitrogen declined with stand age due to high nutrient demand and limited litter input. The available phosphorus and available potassium (AK) contents were identified as key limiting factors, influencing ectomycorrhizal fungi abundance and the overall soil fungal diversity. With an increasing stand age, the fungal diversity decreased, the ectomycorrhizal fungi declined, and the pathogenic fungi increased, exacerbating plantation degradation. Regression analysis further indicated a significant negative correlation between AK content and stand age, suggesting potassium deficiency as a critical driver of tree health decline. This study highlights the pivotal role of soil nutrient availability in shaping rhizosphere fungal communities and sustaining *P. sylvestris* plantations, offering insights into degradation mechanisms and strategies to enhance forest resilience on the Loess Plateau.

## 1. Introduction

The Loess Plateau, spanning approximately 635,000 km^2^ in the upper and middle reaches of the Yellow River, represents the most severely eroded region in both China and the world [1,2]. This pervasive soil erosion has led to profound ecological degradation and has undermined sustainable socioeconomic development [3]. In particular, Youyu County, Shanxi Province, China, located in the northern Loess Plateau, exemplifies an ecologically fragile area that has experienced extensive soil erosion, and has been the focus of large-scale Grain for Green initiatives since the 1970s [4,5].

*Pinus sylvestris* var. *mongholica*, an evergreen conifer renowned for its robust resistance to environmental stress, has been extensively utilized in vegetation restoration and soil erosion mitigation efforts [6]. Consequently, it has been widely planted in Youyu County, playing a pivotal role in local soil conservation [4]. However, in recent years, these plantations have undergone marked degradation, characterized by stunted growth, a diminished resistance to cold, drought, and pest pressures, and a consequent rise in tree mortality. Such deterioration has substantially reduced the plantations’ capacity to control soil erosion. Although previous studies have primarily attributed the degradation of *P. sylvestris* to climatic variability, suboptimal management practices, imbalanced soil water utilization, pest infestations, and high stand density [7,8], these investigations have largely been confined by geographic and spatial limitations and have neglected the potential influence of soil fungi on the degradation process. Thus, the underlying mechanisms driving the degradation of *P. sylvestris* plantations remain incompletely understood.

Soil fungi, a diverse assemblage of microorganisms ubiquitous in terrestrial ecosystems, play a critical role in bridging aboveground and belowground processes [9,10,11]. These fungi exert significant influences on forest health; for instance, saprotrophic fungi accelerate litter decomposition and nutrient cycling in plantation ecosystems [12], while mycorrhizal fungi, through symbiotic associations with tree roots, enhance nutrient uptake and improve growth efficiency [13,14]. Given these functional roles, soil fungi are increasingly recognized as key factors in addressing the degradation of *P. sylvestris* plantations, underscoring the need for a comprehensive understanding of their diversity and community composition.

In monoculture plantations, stand age is a critical determinant influencing the structure and dynamics of the soil fungal communities [15]. Concurrently, the soil physicochemical properties—such as moisture content, pH, and soil organic carbon (SOC)—profoundly affect the diversity and successional trajectories of these communities [16,17]. Therefore, investigating the interplay between the stand age and soil properties in *P. sylvestris* plantations is essential for elucidating the dynamics of the soil fungal communities within the rhizosphere.

In this study, we collected rhizosphere soil samples from *P. sylvestris* plantations of varying stand ages—including degraded forests—in Youyu County. We subsequently analyzed both the soil fungal communities and the associated soil properties across these age classes. The objectives of the study were as follows: (1) elucidate the potential variations in the relationships between the stand age and soil physicochemical properties, (2) investigate the influence of these factors on key fungal communities (e.g., saprotrophic fungi and ectomycorrhizal fungi), and (3) delineate the microecological factors associated with plantation forest degradation in the region.

## 2. Results

### 2.1. Soil Properties

The results of a one-way analysis of variance (ANOVA) showed that the rhizosphere soil properties varied significantly with the stand age (Table 1). Generally, with an increasing stand age, the contents of the soil nutrients exhibited a decreasing trend, except for the pH. Notably, the ammonium nitrogen (AN) and nitrate nitrogen (NN) levels in 25-year-old stands (V25) were significantly higher than those in 20-year-old stands (V20). Conversely, the NN, AN, available phosphorus (AP), and available potassium (AK) contents in 35-year-old stands (V35) were significantly lower than those in V25. Notably, the regression analysis further indicated that AK content decreased significantly with increasing stand age (*p* < 0.05, R^2^ = 0.589).

### 2.2. Composition of Soil Fungal Community

**Figure 1:**
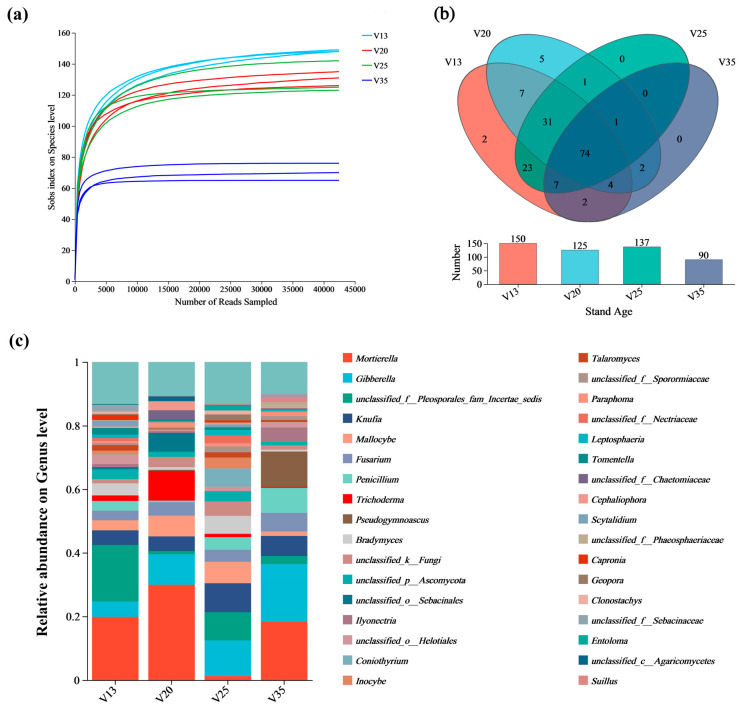
Sequencing results of rhizosphere soil fungi of *P. sylvestris* at different stand ages. (**a**) Rarefaction curves of soil fungi at different stand ages; (**b**) Venn diagram of rhizosphere soil fungal composition of *P. sylvestris* at genus level; (**c**) Composition of rhizosphere soil fungi of *P. sylvestris* at genus level and different stand ages. V13: 13-year-old forest, V20: 20-year-old forest, V25: 25-year-old forest, and V35: 35-year-old forest.

The rarefaction curves of the soil fungi across different stand ages are presented in Figure 1a. The Sobs index at the OTU level remained relatively stable with an increasing number of sequencing reads, indicating that the sequencing depth was sufficient and the dataset was reliable. Following Illumina MiSeq sequencing, a total of 495,708 fungal sequences were obtained, identifying 373 operational taxonomic units (OTUs). Among them, 144 OTUs were classified at the genus level, encompassing 7 phyla, 20 classes, 45 orders, and 92 families. The total number of soil fungal genera exhibited a decreasing trend with increasing stand age (Figure 1b).

In terms of the fungal community composition, the genera *Mortierella*, *Gibberella*, *Knufia*, and *Mallocybe* were relatively abundant (Figure 1c). Specifically, *Mortierella* exhibited a relative abundance of 29.90% in V20, but sharply declined to 1.25% in V25. Conversely, *Gibberella* increased with the stand age, comprising 4.82% in V13, 9.60% in V20, 11.15% in V25, and reaching 17.99% in V35. The relative abundance of *Knufia* remained relatively stable, but increased to 9.08% in V25. Meanwhile, *Trichoderma* accounted for 9.34% in V20, but significantly decreased to 0.33% in V35. These results suggest distinct shifts in the fungal community composition with stand development, highlighting potential ecological changes in the rhizosphere fungal interactions.

### 2.3. Functional Groups of Soil Fungal Community

**Figure 2:**
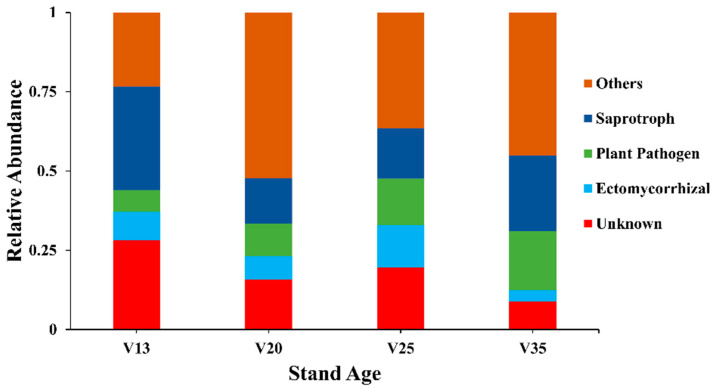
Functional groups of rhizosphere soil fungal communities of *P. sylvestris* at different stand ages. V13: 13-year-old forest, V20: 20-year-old forest, V25: 25-year-old forest, and V35: 35-year-old forest.

The FUNGuild analysis of the soil fungal communities across different stand ages revealed significant variations in functional group composition (Figure 2). Notably, the relative abundance of pathogenic fungi increased markedly with the stand age, peaking at 18.6% in the 35-year-old stands (V35). In contrast, the proportion of ectomycorrhizal fungi declined with the stand age, reaching a minimum of 3.6% in V35. The category labeled ‘other fungi’ exhibited its highest relative abundance in the 20-year-old stands (V20), accounting for 52.3%, while the proportion of saprotrophic fungi was low (14.2%) in the stands of this age.

### 2.4. Diversity of Soil Fungal Community

**Figure 3:**
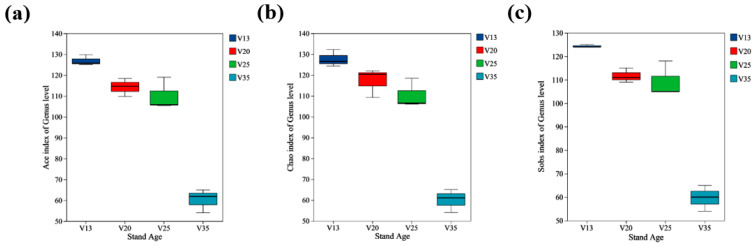
Rhizosphere soil fungal diversity indices of *P. sylvestris* at different stand ages. (**a**) Ace index at genus level; (**b**) Chao index at genus level; (**c**) Sobs index at genus level. V13: 13-year-old forest, V20: 20-year-old forest, V25: 25-year-old forest, and V35: 35-year-old forest.

The Ace index (Figure 3a), Chao index (Figure 3b), and Sobs index (Figure 3c) indicated that the soil fungal community diversity was significantly influenced by the stand age. These diversity indices exhibited a decreasing trend with increasing stand age, with the values in the 35-year-old stands (V35) being significantly lower than those in the younger stands. Additionally, no significant differences were observed in the Sobs and Ace indices between the 20-year-old (V20) and 25-year-old (V25) stands, suggesting that the fungal diversity remained relatively stable within this age range.

### 2.5. Relationship Between Soil Fungal Community Composition and Soil Physicochemical Properties

**Figure 4:**
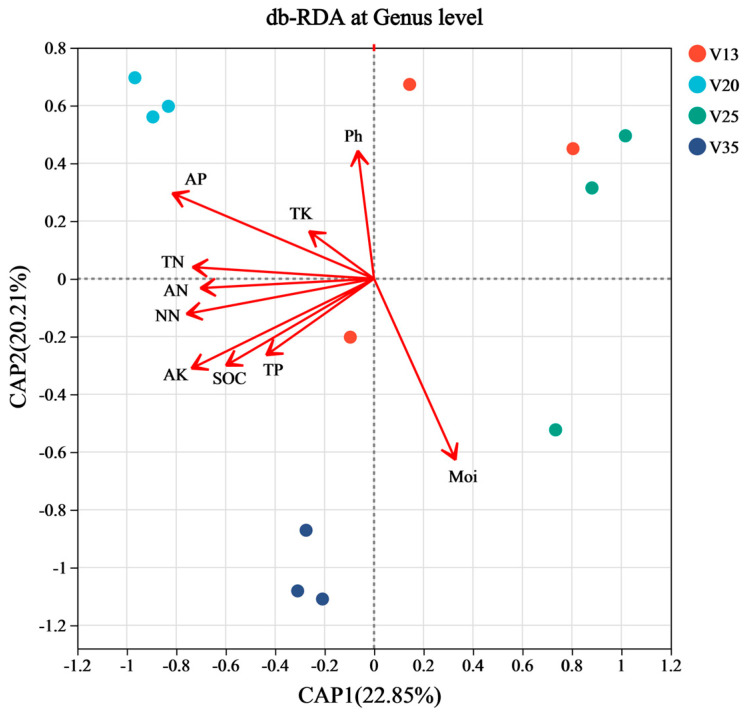
Distance-based redundancy analysis (db-RDA) of rhizosphere soil fungal community structure of *P. sylvestris* at different stand ages. TN: total nitrogen, NN: nitrate nitrogen, AN: ammonium nitrogen, TP: total phosphorus, AP: available phosphorus, TK: total potassium, AK: available potassium, SOC: soil organic carbon, Moi: soil moisture content, V13: 13-year-old forest, V20: 20-year-old forest, V25: 25-year-old forest, and V35: 35-year-old forest.

The results of a distance-based redundancy analysis (db-RDA) indicated that the soil physicochemical properties were significantly correlated with the soil fungal community structures across different stand ages (Figure 4). Specifically, the fungal community structure in V20 was positively correlated with the contents of AP, AN, NN, SOC, and AK. Meanwhile, the fungal community structure in V35 showed a positive correlation with AK, SOC, and Moi. Other soil properties did not exhibit significant effects on the fungal community structure.

**Figure 5:**
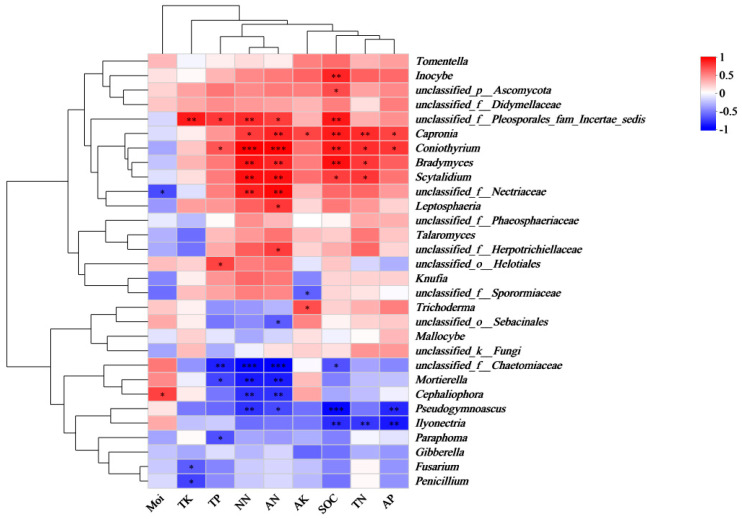
Correlation heatmap of rhizosphere soil fungal genera and soil properties of *P. sylvestris*. TN: total nitrogen, NN: nitrate nitrogen, AN: ammonium nitrogen, TP: total phosphorus, AP: available phosphorus, TK: total potassium, AK: available potassium, SOC: soil organic carbon, Moi: soil moisture content, *: significant at *p* < 0.05, **: significant at *p* < 0.01, and ***: significant at *p* < 0.001.

The correlation analysis between the relative abundance of fungal genera from different functional groups and the soil properties (Figure 5) revealed distinct associations. Ectomycorrhizal fungi, including *Tomentella*, *Inocybe*, and *Mallocybe*, were positively correlated with AK, TK, and AP. In contrast, the pathogenic fungus *Gibberella* was negatively correlated with Moi, TP, NN, AN, TK, AP, SOC, TN, and AK, showing a particularly strong negative correlation with TK. *Leptosphaeria* exhibited a negative correlation with Moi, but a positive correlation with TP, NN, AN, TK, AP, SOC, TN, and AK. Regarding saprophytic fungi, *Talaromyces* and *Scytalidium* displayed a positive correlation with TP, NN, AN, AP, SOC, TN, and AK, while being negatively correlated with Moi. On the other hand, *Trichoderma*, *Ilyonectria*, and *Penicillium* were negatively correlated with TP, NN, and AN.

**Figure 6:**
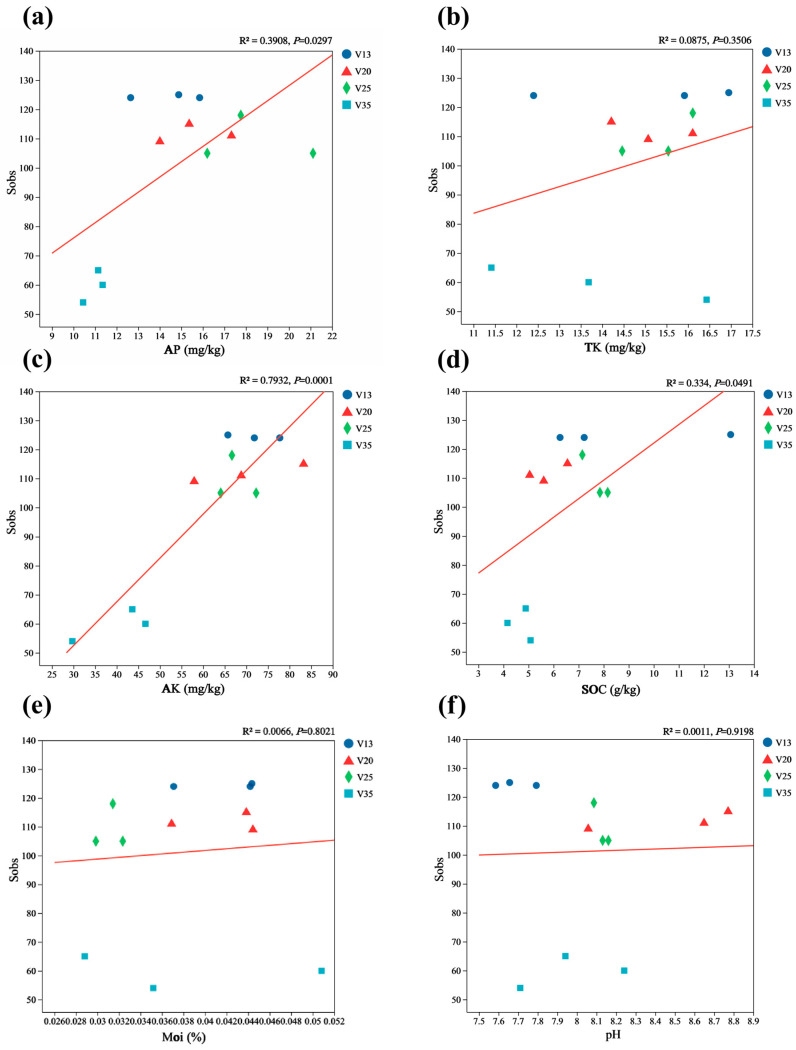
The correlation of the rhizosphere soil fungal Sobs index with the soil properties of *P. sylvestris* at different stand ages. (**a**) AP: available phosphorus; (**b**) TK: total potassium; (**c**) AK: available potassium; (**d**) SOC: soil organic carbon; (**e**) Moi: soil moisture content; (**f**) pH. The level of fit and the significance of the relationship is shown in the top right corner of each panel. V13: 13-year-old forest, V20: 20-year-old forest, V25: 25-year-old forest, and V35: 35-year-old forest.

Furthermore, the regression analysis demonstrated a significant linear relationship between the Sobs index and AK content (Figure 6c), suggesting that available potassium plays a crucial role in shaping soil fungal diversity. However, the TN, NN, AN, TP, AP, TK, Moi, pH, and SOC contents did not exhibit significant correlations with the Sobs index.

## 3. Discussion

The survival and growth of *P. sylvestris* depend on soil moisture and nutrient availability. Previous studies have shown that as stand age increases, *P. sylvestris* promotes local carbon (C) and nitrogen (N) cycling, thereby enhancing soil C and N storage [18]. However, in this study, the SOC and TN contents in the rhizosphere soil of *P. sylvestris* significantly decreased with the stand age. This phenomenon may stem from the early growth stage of plantations, where *P. sylvestris* has high carbon and nitrogen demands, but a limited capacity to fix them [19,20]. Additionally, litter accumulation, which contributes to soil C and N storage, has not reached sufficient levels [21,22]. Over time, the soil nutrients gradually decrease. Notably, a recovery trend in the soil nutrients was observed in mature plantations. For example, the SOC, TN, AN, and NN contents were significantly higher in V25 than in V20. This may be attributed to the increased stand density in V25, which promotes litter accumulation and enhances soil C and N storage. Furthermore, the reduced nutrient demands of mature plantations may also contribute to soil nutrient recovery [23].

The Sobs, Ace, and Chao diversity indices of the soil fungal community decreased with increasing stand age. This aligns with previous findings, indicating that an increasing stand age significantly reduces the diversity of rhizosphere soil fungal communities [24]. A long-term monoculture plantation pattern can exacerbate imbalances in the rhizosphere micro-ecosystem, significantly reducing the diversity and metabolic activity of the soil microbial communities [25]. During sampling, we observed a gradual decrease in the biomass and species richness of understory herbs with increasing stand age, which is consistent with previous studies. Understory herbs play a crucial role in maintaining soil microbial communities by producing litter that provides physical protection for the microbes and affects the soil properties, which serve as key resources for microbial growth [26,27,28]. Therefore, the reduction in understory herbs may be a key factor contributing to the decline in soil fungal diversity. As stand age increases, the degradation of the pine plantations results in decreased stand density, and trees serve as the primary source of soil litter [29,30]. Since most soil microbes are saprophytic and rely on litter for growth, a reduction in stand density leads to a decrease in litter quantity, negatively impacting the soil fungal diversity. Although the canopy thinning in older stands might theoretically improve light conditions and promote the growth of understory herbs, our field observations revealed a consistent decline in the understory biomass and species richness with increasing stand age. This suggests that light availability alone may not be sufficient to stimulate understory regeneration, particularly under conditions of declining soil fertility and increasing tree root competition. Consequently, the reduction in tree litter input was not compensated for by herbaceous vegetation, leading to an overall decline in litter contribution to the soil and, in turn, indirectly reducing the soil fungal community diversity.

Soil properties are closely related to the distribution and diversity of soil fungal communities [31]. In this study, we found a significant correlation between the soil properties and the composition and diversity of the soil fungal communities. *Mortierella* and *Gibberella* were the two most abundant genera in the soil fungal community. Previous studies have indicated that *Mortierella* is a core keystone taxon at different growth stages of *P. sylvestris* [32], while *Gibberella* is a common pathogenic genus [33,34]. In our study, the relative abundances of *Mortierella* and *Gibberella* were negatively correlated with SOC, TN, AN, NN, AP, TP, and TK contents. As stand age increases, these soil properties decrease, maintaining the dominance of these fungal genera in the community. The increasing relative abundance of *Gibberella* with stand age further supports this conclusion.

Moreover, in V25, the relative abundance of *Mortierella* dramatically decreased, losing its dominance, while *Knufia*, which was positively correlated with SOC, TN, AN, NN, and AP contents, significantly increased. This shift may be attributed to the significant increase in the SOC, TN, AN, NN, and AP contents in V25 compared to V20 (even exceeding those in V13). Additionally, the number of fungal genera was higher in V25 than in V20, which we speculate is due to the substantial increase in soil nutrients. Soil physicochemical properties and nutrient availability are crucial limiting factors for fungal growth, and profoundly influence the composition of soil fungi [35,36]. Therefore, the recovery of the soil physicochemical properties in V25 provided favorable conditions for the restoration of the soil fungi [18].

Meanwhile, microbial communities are key shapers of the rhizosphere microenvironment [37]. The functional groups of soil fungal communities, particularly the ectomycorrhizal fungi and plant pathogenic fungi, significantly impact tree growth [38]. Previous studies have shown that in healthy *P. sylvestris* plantations, the diversity of the ectomycorrhizal fungi in the rhizosphere soil increases with stand age, promoting nutrient absorption and providing physical protection to the roots [39,40]. However, in our study, the relative abundance of ectomycorrhizal fungi in the soil fungal community decreased with stand age, weakening the resistance of the pine trees to stresses such as cold and drought [41] and increasing the risk of pests like *Bursaphelenchus xylophilus* [42]. Meanwhile, the relative abundance of pathogenic fungi increased significantly with stand age, raising the risk of disease infection. Pathogenic fungi such as *Sphaeropsis sapinea* can cause stress-induced diseases such as shoot blight and canker disease, damaging the wood and phloem of infected trees and producing toxic metabolites that result in tree death [43,44,45]. Therefore, the decrease in ectomycorrhizal fungi and the increase in pathogenic fungi with stand age may collectively result in habitat deterioration, accelerating plantation degradation.

Notably, ectomycorrhizal fungi such as *Tomentella, Mallocybe,* and *Inocybe* were positively correlated with AP content, while pathogenic fungi such as *Gibberella* were negatively correlated with AP content. Therefore, supplementing the local soil with phosphate fertilizer may relieve P stress in *P. sylvestris* and improve the functional composition of the soil fungal community, aiding in plantation disease management. Additionally, the AP content in the degraded plantations (V35) was significantly lower than in the normal plantations (V13, V20, V25). Cao et al. (2024) found that AP content is significantly negatively correlated with plant disease incidence, and under adequate P levels, plant disease-suppressive genes increase significantly during pathogen infection. Therefore, increasing the AP content in the soil may help relieve survival stress in *P. sylvestris*.

Furthermore, we speculate that the increasing stand age, soil potassium (K) stress, and changes in the soil fungal functional groups led to a significant deterioration of the rhizosphere microenvironment, profoundly impacting *P. sylvestris* degradation. The linear regression analysis indicated a significant negative correlation between AK content and stand age. K is an essential nutrient for tree growth, playing a key role in regulating physiological processes, particularly in alleviating drought stress [46,47]. K stress weakens the metabolic activity and growth ability of pine trees. For instance, K deficiency reduces lateral root elongation in pines, impairing nutrient and water uptake [48], and causes needle yellowing [49]. During sampling, we observed increasing needle yellowing with stand age, and in some plots, entire needles turned yellow. Additionally, older pine trees had a significantly reduced lateral root biomass. These observations are consistent with K stress, suggesting that K deficiency may be a critical driver of *P. sylvestris* degradation in this region.

Although our findings emphasized the key roles of AP and AK, we acknowledge that the nitrogen indices (TN, AN, NN) also exhibited pronounced fluctuations and ecological significance. Specifically, the declines in TN, AN, and NN observed in the older plantations (V35) suggest a broader pattern of diminished microbial mineralization capacity and nutrient turnover. As nitrogen is critical for microbial biomass and function, the decreased nitrogen availability likely contributes indirectly to the shifts in fungal community composition and plantation degradation. This phenomenon warrants further investigation in future studies.

It is worth noting that, although this study focused solely on the rhizosphere soil and excluded the surface litter layer during sampling, the quantity and composition of the litter likely influence long-term nutrient cycling and fungal community development. Future studies may benefit from integrated sampling of both litter and soil to better capture the aboveground–belowground interactions.

## 4. Conclusions

This study reveals that the *P. sylvestris* rhizosphere soil nutrients, particularly the SOC and TN, declined with stand age due to high nutrient demands and limited litter accumulation. However, the mature plantations exhibited nutrient recovery, likely due to an increased stand density. The soil fungal diversity decreased with stand age, driven by the understory herb loss and reduced litter input. The ectomycorrhizal fungi declined, weakening tree resistance, while pathogenic fungi like *Gibberella* increased, raising disease risks. *Mortierella* declined in the mature stands, while *Knufia*, associated with higher nutrients, became dominant. Available phosphorus (AP) and potassium (AK) were critical for soil health. The AP supported the ectomycorrhizal fungi and suppressed pathogens, while AK deficiency contributed to *P. sylvestris* degradation. Given these findings, we preliminarily recommend phosphorus and potassium fertilization, appropriate stand density management, and the promotion of understory vegetation as potential strategies to mitigate the degradation of *P. sylvestris* plantations. However, their long-term effectiveness and ecological impact should be further validated through multi-site and longitudinal studies.

## 5. Materials and Methods

### 5.1. Study Site Description

The study was conducted in Youyu County, Shuozhou City, Shanxi Province, China (coordinates: 40°25′ N, 112°32′ E). The sampling site is situated at the northwestern edge of Shanxi Province, on the periphery of the Loess Plateau. The region exhibits an average annual precipitation of 319.5 mm and an average annual temperature of 13 °C. Characterized as a gentle-slope hilly sandstorm area, Youyu County has an average elevation of approximately 1400 m. The local climate is marked by strong winds, abundant sand, low temperatures, limited rainfall, and severe wind and water erosion, all of which contribute to a highly degraded ecological environment. With a total area of 1969 km^2^, approximately 1498.85 km^2^ of Youyu County is affected by soil erosion, underscoring its critical role in regional soil and water loss control on the Loess Plateau.

The selected plantation stands were established under similar initial conditions. The study sites were all located on gentle slopes (less than 5° gradients) with comparable aspects and elevations to minimize the variations caused by topography. The soil type across all selected stands was a typical loessial soil, characterized by uniform parent materials (loess deposits), textures (predominantly silt loam), and similar initial nutrient levels.

The initial planting densities across the selected plantations were standardized according to the local forestry management guidelines, typically adopting a spacing of approximately 2 m × 3 m (around 1650 trees per hectare). While minor variations in the planting density might exist due to historical management practices, such differences were within the normal operational range and unlikely to significantly affect the comparability of the sites. Moreover, the sampling plots within each age class were carefully chosen to be representative of the typical stand conditions, thereby minimizing the potential biases arising from any initial planting discrepancies.

These plantations were established as part of local ecological restoration initiatives, including the afforestation of barren slopes and forest regeneration projects. Consequently, extensive areas of *P. sylvestris* plantations at varying stand ages now exist throughout the study region. However, these plantations currently exhibit differing degrees of degradation, making this area ideal for examining the factors influencing forest health and sustainability.

### 5.2. Sample Collection

This study employed a time–space substitution approach by selecting *P. sylvestris* plantations representing four stand ages (13, 20, 25, and 35 years) as the research subjects. All sampling sites were located within the same ecological region and shared similar topographic features and loessial soil conditions. The distance between plantations of different age classes ranged from approximately 0.5 to 2 km, depending on the site availability and accessibility.

For each age class, three independent sampling plots (15 m × 15 m) were established within a single stand and spaced at least 50 m apart to ensure plot independence while maintaining the environmental consistency. Within each plot, a five-point composite sampling method was applied: rhizosphere soil (sensu lato) was collected from five healthy and representative trees located at the four corners and the center of each plot. For each tree, the soil was excavated from the root zone (approximately 1 m from the trunk) in four directions (east, west, south, and north) to a depth of 40 cm. The subsamples were first combined at the tree level, and then all five tree-level samples were mixed to generate one composite soil sample per plot, resulting in three biological replicates per stand age.

Each composite sample was divided into two subsamples: one was immediately flash-frozen in liquid nitrogen and stored at −80 °C for soil fungal community analysis, while the other was air-dried and used for the determination of the soil physicochemical properties. To ensure consistency, only trees with typical canopy structures, heights, and diameters at breast height (DBHs) were selected, while those exhibiting disease symptoms, mechanical damage, or abnormal growth were excluded.

The understory vegetation was primarily composed of native herbaceous species such as *Artemisia capillaris* and *Setaria viridis*. Field observations showed that both the understory coverage and the species richness declined with increasing stand age, with the 35-year-old stands exhibiting noticeably sparse ground vegetation.

Although the soil erosion was not quantitatively measured, qualitative field observations indicated minor signs of erosion—such as surface runoff traces, shallow root exposure, and localized soil crusting—primarily in the older plantations. However, no severe erosion features (e.g., gully erosion) were observed.

All field sampling was conducted in August, during the local peak growing season, which ensured high microbial activity and minimized the influence of seasonal variation on the soil nutrient levels and fungal community composition.

### 5.3. Analysis of Soil Samples

The total nitrogen (TN) and nitrate nitrogen (NN) were determined using the Kjeldahl method and UV spectrophotometry, respectively [50]. Ammonium nitrogen (AN) was quantified via a colorimetric method [51]. The soil organic carbon (SOC) was measured by dichromate oxidation [52]. The total phosphorus (TP) was assessed using molybdenum antimony blue colorimetry [53], while the available phosphorus (AP) was extracted with sodium bicarbonate and analyzed spectrophotometrically [54]. The total potassium (TK) was determined after digestion with hydrofluoric and perchloric acids, and the available potassium (AK) was extracted using 1 N ammonium acetate and quantified by atomic absorption spectroscopy [18]. The soil moisture content was evaluated via thermogravimetric analysis [55], and the soil pH was measured using colorimetric methods [56].

### 5.4. Total DNA Extraction from Rhizosphere Soil Samples

The soil samples after pure culture were weighed, and the total DNA was extracted from the soil with an EZNA Soil DNA Kit (D5625-01, Omega, Macon, GA, USA) [57]. To verify successful extraction, agarose gel electrophoresis with a 1% concentration was employed for DNA separation. The concentration and purity of the DNA were assessed using a NanoDrop 2000 UV–vis spectrophotometer (Thermo Fisher Scientific, Wilmington, NC, USA), 1 µL DNA solution was used to measure the absorbance, and the data were recorded [58]. The quality of the total DNA was detected by agarose gel electrophoresis; 3 µL DNA solution was evenly mixed with 0.5 µL loading buffer (10×) and added into 1% agar gel for electrophoresis under the following conditions: 1 × TAE electrophoresis buffer (20 mM Tris, 10 mM Acetate, and 0.5 mM Na_2_EDTA, pH 8.0) at 130 V for 15 min. The total DNA extracted above was amplified by PCR using the fungi universal primers ITS1-F (5′-CTTGGTCATTTAGAG-GAAGTAA-3′) and ITS2-R (5′-CTTGGTCATTTAGAGGAAGTAA-3′). The volume for the PCR was 20 µL, containing 10 μL of PCR SuperMix (TransGen Biotech, Beijing, China), template DNA (1 µL), 0.5 μL of each primer, and 8 μL of ddH_2_O [59]. The PCR amplification was carried out as follows: initial denaturation at 95 °C for 5 min, followed by 30 cycles of denaturation at 95 °C for 30 s, annealing at 55 °C for 30 s, and extension at 72 °C for 1 min. After completing all cycles, a final extension was performed at 72 °C for 5 min. The PCR was carried out in triplicate. Agarose gel electrophoresis was used to detect the amplified fragments: 3 µL PCR product was loaded onto a 1% agarose gel and electrophoresed at 130 V for 15 min in 1 × TAE buffer. The PCR products were then sent to Shanghai Majorbio Bio-Pharm Technology Co., Ltd. (Shanghai, China) (https://www.majorbio.com) for Illumina MiSeq sequencing.

### 5.5. Data Processing

The raw sequencing data from Illumina MiSeq were quality-controlled using fastp (Version 0.20.0; https://github.com/OpenGene/fastp, accessed on 12 May 2023) and assembled with FLASH (Version 1.2.7; http://www.cbcb.umd.edu/software/flash, accessed on 19 May 2023). The operational taxonomic units (OTUs) were clustered at a 97% similarity threshold, and chimeric sequences were removed using UPARSE (Version 7.1; http://drive5.com/uparse/, accessed on 24 May 2024). The taxonomic assignments were performed by comparing individual sequences against the UNITE fungal ITS database (Version 8.0) using the RDP Classifier (Version 2.2; http://rdp.cme.msu.edu/, accessed on 25 May 2024). The functional categorization of the fungal sequences, particularly for the identification of the ectomycorrhizal fungi, was achieved using the FUNGuild database (https://github.com/UMNFuN/FUNGuild, accessed on 17 July 2024).

### 5.6. Statistical Analysis

The statistical significance of the differences in the soil properties and soil fungal diversity and composition among the stand ages was determined by a one-way analysis of variance (ANOVA) using SPSS (Version 25). Prior to conducting the ANOVA and regression analysis, the data were tested for normality using the Shapiro–Wilk test and for homogeneity of variance using Levene’s test. These tests confirmed that the data met the assumptions required for the parametric analyses. Duncan multiple range tests were performed to assess the statistically significant differences between the values at a 95% confidence level. The Venn diagram was drawn by using R software (Version 3.3.1, Vegan package). Alpha diversity (α-diversity) was used to examine the complexity of the species diversity in the sample using three indices (Ace, Chao, and Sobs), which were used to analyze the fungal community diversity, and the rarefaction curves were drawn. A distance-based redundancy analysis (db-RDA) was used to investigate the importance of environmental factors in explaining the distribution patterns of the fungal communities for different stand ages using R software (Version 4.0.4, Vegan package). A heatmap correlation analysis was performed to evaluate the relationships between the soil properties and the soil fungal communities using R software (Version 4.0.4, Pheatmap package). The interactions between the Sobs index and soil properties were analyzed by the regression analysis using R software (Version 4.0.4).

## Figures and Tables

**Table 1:** The rhizosphere soil properties of *P. sylvestris* at different stand ages.

Stand Age	TN (mg/kg)	NN (mg/kg)	AN (mg/kg)	TP (mg/kg)	AP (mg/kg)	TK (g/kg)	AK (mg/kg)	SOC (g/kg)	Moi (%)	pH
V13	217.66 ± 21.03 ab	2.45 ± 0.23 ab	12.85 ± 0.62 b	202.89 ± 33.39 a	14.46 ± 1.64 b	15.09 ± 2.39 a	71.77 ± 6.01 a	8.85 ± 3.69 a	0.0419 ± 0.0042 a	7.68 ± 0.11 b
V20	213.27 ± 31.39 ab	1.45 ± 0.35 c	7.30 ± 1.08 d	156.70 ± 10.19 b	15.57 ± 1.67 ab	15.13 ± 0.95 a	69.99 ± 12.71 a	5.74 ± 0.76 ab	0.0417 ± 0.0042 a	8.49 ± 0.38 a
V25	261.90 ± 52.71 a	2.86 ± 0.29 a	15.14 ± 0.56 a	195.24 ± 19.36 ab	18.36 ± 2.51 a	15.37 ± 0.84 a	67.67 ± 4.19 a	7.72 ± 0.52 ab	0.0312 ± 0.0013 a	8.13 ± 0.04 ab
V35	180.80 ± 26.93 b	1.97 ± 0.13 b	10.93 ± 1.29 c	174.43 ± 14.19 ab	10.98 ± 0.48 c	13.84 ± 2.51 a	39.99 ± 9.01 b	4.71 ± 0.49 b	0.0383 ± 0.0113 a	7.96 ± 0.27 b
*p*-value	ns	ns	ns	ns	ns	ns	Linear Regression (*p* < 0.05, R^2^ = 0.589)	ns	ns	ns

Data expressed as means ± standard deviations (n = 3). TN: total nitrogen, NN: nitrate nitrogen, AN: ammonium nitrogen, TP: total phosphorus, AP: available phosphorus, TK: total potassium, AK: available potassium, SOC: soil organic carbon, Moi: soil moisture content. V13: 13-year-old forest, V20: 20-year-old forest, V25: 25-year-old forest, and V35: 35-year-old forest. Lowercase letters indicate significant differences, with *p*-values < 0.05 based on analysis of variance; “ns” indicates regression analysis results were not significant (*p* ≥ 0.05).

## Data Availability

The raw data supporting the conclusions of this article will be made available by the authors on request.
